# 1071. Retrospective study evaluating the cytokine dynamics in COVID-19 patients who were treated with casirivimab/imdevimab

**DOI:** 10.1093/ofid/ofac492.912

**Published:** 2022-12-15

**Authors:** Akiho Sugita, Masahiro Ishikane, Noriko Iwamoto, Masaya Sugiyama, Katsuji Teruya, Masayuki Hojo, Masashi Mizokami, Norio Ohmagari

**Affiliations:** Center Hospital of the National Center for Global Health and Medicine, Tokyo, Tokyo, Japan; Center Hospital of the National Center for Global Health and Medicine, Tokyo, Tokyo, Japan; Disease Control and Prevention Center, Tokyo, Tokyo, Japan; Research Institute, Center Hospital of the National Center for Global Health and Medicine, Tokyo, Tokyo, Japan; Center Hospital of the National Center for Global Health and Medicine, Tokyo, Tokyo, Japan; Center Hospital of the National Center for Global Health and Medicine, Tokyo, Tokyo, Japan; Center Hospital of the National Center for Global Health and Medicine, Tokyo, Tokyo, Japan; National Center for Global Health and Medicine, Tokyo, Tokyo, Japan

## Abstract

**Background:**

Neutralizing antibody therapy such as casirivimab/imdevimab is known to significantly reduce the viral load of SARS-CoV-2, but there is limited study on the clinical prognosis of neutralizing antibody therapy, especially in Asia, and the dynamics of cytokines is unknown worldwide. Several cytokines have been investigated as biomarkers to predict oxygen demand, among which CCL17 and INF3 have received approved and covered by the national health insurance in Japan.

**Methods:**

Between July 2021 - December 2021, patient’s demographic, laboratory, radiological findings, prognosis, and cytokine kinetics (IFN-λ3, CCL17) at National Center for Global Health and Medicine, Tokyo, Japan, were analyzed using medical charts and serum samples. Univariate analysis was performed using Fisher's exact probability test and Mann-Whitney U test to evaluate the clinical characteristics of the group with oxygen demand compared with those of the group without oxygen demand.

**Results:**

Thirty-four patients were analyzed. The median age of the cohort was 57.5 years (IQR 52.8-67.3), and 25 (73.5%) were male. Eight patients (23.5%) had been fully vaccinated and three patients (8.8%) had been vaccinated once. The severity of disease before casirivimab/imdevimab was asymptomatic in two (5.9%), mild in 12 (35.3%), moderate in 20 (58.8%) cases. Of the 17 cases in which mutant strains were identified, 16 were delta strains. The IFN-λ3 level (pg/mL) before casirivimab/imdevimab was significantly higher (7.6 vs. 17.2, *p* = 0.005), while the CCL17 level (pg/mL) was significantly lower (148.8 vs. 64.2, *p* = 0.036) in the group with oxygen demand during the therapeutic course compared to those in the group without oxygen demand. After casirivimab/imdevimab was administered, the IFN-λ3 level decreased to a median of 0.0 (IQR 0.0-0.3), while the CCL17 level increased to median of 220.3 (IQR 135.8-304.8), with no statistically significant differences between both groups (Figure 1). None of the patients became seriously ill.

**Figure:**
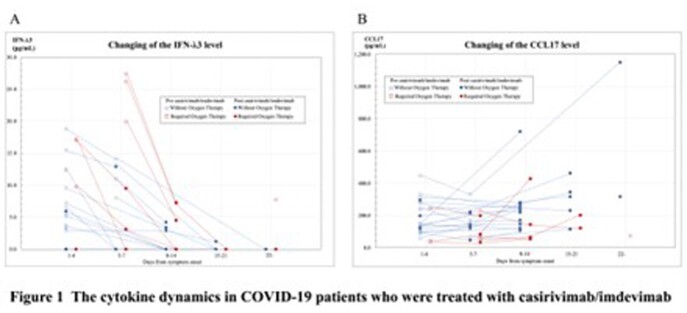

Figure 1A and 1B show the changing of the cytokine dynamics in COVID-19 patients who were treated with casirivimab/imdevimab on IFN-λ3 level and CCL17 level, respectively.

**Conclusion:**

There was a statistically significant difference between IFN-λ3 and CCL17 levels before casirivimab/imdevimab in both groups. Our results suggest that casirivimab/immudevimab may improve the clinical prognosis for COVID-19 patients with delta strains.

**Disclosures:**

**All Authors**: No reported disclosures.

